# The Modified Atkins Diet in Refractory Epilepsy

**DOI:** 10.1155/2014/404202

**Published:** 2014-01-30

**Authors:** Suvasini Sharma, Puneet Jain

**Affiliations:** Division of Pediatric Neurology, Department of Pediatrics, Lady Hardinge Medical College and Associated Kalawati Saran Children's Hospital, New Delhi 110001, India

## Abstract

The modified Atkins diet is a less restrictive variation of the ketogenic diet. This diet is started on an outpatient basis without a fast, allows unlimited protein and fat, and does not restrict calories or fluids. Recent studies have shown good efficacy and tolerability of this diet in refractory epilepsy. In this review, we discuss the use of the modified Atkins diet in refractory epilepsy.

## 1. Introduction

Seizures are a frequent cause of morbidity in the pediatric age group [1]. Several severe catastrophic epilepsies present in childhood, including severe infantile myoclonic epilepsy, West syndrome, Lennox-Gastaut syndrome, and myoclonic-astatic epilepsy (Doose syndrome) [2]. Seizures in these epilepsy syndromes are difficult to control, with the added problems of multiple and toxic levels of antiepileptic medications [3]. Epilepsy surgery may not work in these patients and also the costs are prohibitively high. Uncontrolled seizures pose a variety of risks to children, including higher rates of mortality, developmental delay and/or regression, and cognitive impairment [4]. The shortcomings of antiepileptic drug therapy and epilepsy surgery have made the need for alternative treatments.

The ketogenic diet is one of the oldest available treatments for epilepsy. It is a medically supervised high fat, low carbohydrate, and restricted protein diet that maintains a chronic state of ketosis. The ketogenic diet compares favourably with the newer antiepileptic drugs (AEDs) which have been developed for the treatment epilepsy in children [5, 6]. With the ketogenic diet, 33% of patients with intractable epilepsy have more than 50% reduction in seizures and 15–20% become seizure free [7–11]. Also, many of the children who are maintained on the diet are able to have their antiepileptic drugs decreased or withdrawn. This leads to improvement in alertness, behavior, and cognition.

The traditional ketogenic diet, with 4 : 1 ratio of fat: carbohydrate + protein, has its drawbacks. It restricts calories and fluids and requires strict weighing of foods. Protein is generally restricted, with the majority of the remaining calories in the form of fat. This may lead to hypoproteinemia and growth problems. Hospitalization is generally advocated for diet initiation. Side effects of the diet include kidney stones, constipation, acidosis, diminished growth, weight loss, and hyperlipidemia [7–11].

The modified Atkins diet is a less restrictive alternative to the traditional ketogenic diet [12, 13]. This diet is started on an outpatient basis without a fast, allows unlimited protein and fat, and does not restrict calories or fluids [12–14]. In this review we discuss the use of the modified Atkins diet in refractory epilepsy.

## 2. The Atkins Diet

**Figure 1:**
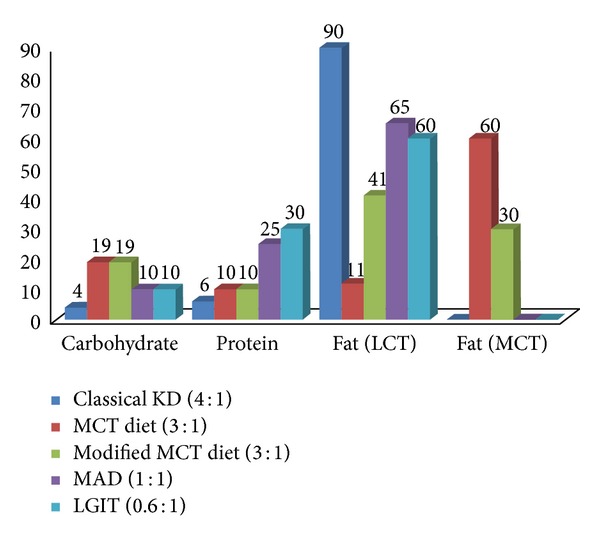
Composition of the ketogenic diets and their variants.

The Atkins diet was developed in the United States in 1970 by Robert C. Atkins for the purpose of weight loss. This diet allowed the intake of fat and the restriction of carbohydrates. The modified Atkins diet is “*modified*” from the Atkins diet as the “induction phase” of the diet limiting carbohydrates is maintained indefinitely, fat is encouraged (not just allowed), and seizure control is the goal rather than weight loss [15]. In contrast to the ketogenic diet, it does not restrict protein intake or daily calories. The Atkins diet allows meals containing 60% fat, 30% protein, and 10% carbohydrates [16].  Figure 1 shows the composition of MAD as compared to other diets. Because of strong carbohydrate restriction, patients following the Atkins diet also produce ketones [17].

## 3. Use of the Modified Atkins Diet in Refractory Epilepsy

With its comparatively fewer dietary restrictions, the Atkins diet may be less restrictive than the ketogenic diet. Kossoff et al. hypothesized that the Atkins diet can induce metabolic ketosis and might reduce seizures in patients with epilepsy, similar to the ketogenic diet [12]. In 2003, they published a pilot study of six patients, aged 7 to 52 years, who were started on the Atkins diet for the treatment of intractable focal and multifocal epilepsy [12]. Five patients maintained moderate to large ketosis for periods of 6 weeks to 24 months; three patients had seizure reduction and were able to reduce antiepileptic medications. This study provided preliminary evidence that the Atkins diet may have a role as therapy for patients with medically resistant epilepsy.

The same group published their experience with the modified Atkins diet in 20 children with intractable epilepsy in 2006 [13]. Eighty percent of the patients stayed on the diet for six months. In all children, at least moderate urinary ketosis developed within 4 days of starting the modified Atkins diet. At 6 months on the diet, 65% had a >50% response, and 35% had a >90% response. This was strikingly similar to prospective studies on the traditional ketogenic diet [8].

Kang et al. evaluated the efficacy, safety, and tolerability of the modified Atkins diet in fourteen Korean children with refractory epilepsy [14]. Six months after diet initiation, seven (50%) remained on the diet, five (36%) had >50% seizure reduction, and three (21%) were seizure free. The diet was well tolerated by 12 (86%) patients, despite the fact that this was a predominantly rice eating population.

The ideal starting carbohydrate limit in the modified Atkins diet is not yet established. Preliminary studies used 10 grams of carbohydrates/day. However, in one of these studies, 10 (50%) of 20 children had increased carbohydrates to 15 g per day after the first month, and one child increased to 20 g per day after the fourth month, as assessed by the dietary chart reviews [13]. Only one of these children reported a reduction in efficacy as a result, and none described decreased levels of urinary ketosis. Based on these results, Kossoff et al. performed a study to determine the initiating carbohydrate limits in the modified Atkins diet [18]. Twenty children with intractable epilepsy were randomized to either 10 or 20 g of carbohydrates per day for the initial 3 months of the modified Atkins diet and then crossed over to the opposite amount. A significantly higher likelihood of >50% seizure reduction was noted for children started on 10 g of carbohydrate per day at 3 months: 60% versus 10% (*P* = 0.03). Most parents reported no change in seizure frequency or ketosis between groups but improved tolerability with 20 g per day. The authors concluded that a starting carbohydrate limit of 10 g per day for children starting the modified Atkins diet may be ideal, with a planned increase to a more tolerable 20 g per day after 3 months.  Table 1 summarizes the pediatric studies of MAD.

In a recent randomized controlled trial [28], 102 children aged 2 to 14 years who had daily seizures despite the appropriate use of at least 3 antiepileptic drugs were randomized to receive either the modified Atkins diet (*n* = 50) or no dietary intervention (*n* = 52) for a period of three months. The on-going antiepileptic medications were continued unchanged in both groups. Four patients discontinued the diet before the study endpoint, and three patients in the control group were lost to follow up. The median seizure frequency at 3 months, expressed as a percentage of the baseline, was significantly less in the diet group (37.3% versus 100%, *P* = 0.003). The proportion of children with >90% seizure reduction (30% versus 7.7%, *P* = 0.005) and >50% seizure reduction was significantly higher in the diet group (52% versus 11.5%, *P* < 0.001). Constipation was the commonest adverse effect (46%) among children on the diet.

Most of the studies have reported short term seizure outcome following diet initiation. Recently, Chen and Kossoff [34] reported long term follow-up of 87 MAD-treated children. Fifty-four children continued diet beyond 6 months. After a mean of 19.9 months on diet, 30/54 (55%) children with diet durations of >6 months achieved >50% improvement and 19 (35%) were seizure-free. The adverse effects were predominantly elevations in lipid profile and gastrointestinal upset.

This diet has the advantages of nonfasting initiation. Also, it can be used in resource constraint settings with limited dietician support, as it does not require tedious calculations [35]. The counseling time is reduced to 30–60 minutes. In the West, this diet has predominantly been advocated for adolescents and adults. However, in India, this diet has been found useful in young children as well. In a study of 15 children, aged 6 months to 3 years, with infantile spasms refractory to hormonal therapy and/or vigabatrin, the modified Atkins diet was found to render 6 children (40%) spasm free with EEG resolution of hypsarrhythmia at 3 months [26]. The diet was well tolerated in these young children.

Further, the efficacy of the ketogenic diet can be maintained when switching to MAD [12]. The information regarding additional seizure control when switching from MAD to ketogenic diet is limited. In one retrospective review, 37% of patients (10/27) had ≥10% additional seizure reduction with the KD over the MAD with most favorable response seen in children with myoclonic astatic epilepsy [36].

## 4. MAD in Adults

Three open-label studies have reported use of the MAD exclusively in adults [30, 31, 33]. Three studies have reported on adolescents in mixed cohorts of children and adolescents [14, 20, 32]. One case series has included one adolescent and three adults [12]. Data from six studies show that, on average, 18 of 66 (27%) adolescents and adults achieved ≥50% seizure reduction; of these 66 individuals, 1 (6%) became seizure-free. Treatment may be slightly more effective in those with higher initial seizure frequencies and in younger adults.

One study found that, after an average of 3 days, all adults had positive results for urinary ketones, both in the morning and evening [31]. In another study, all 28 adults who remained on the diet for at least 1 week became ketotic, but only 2 of 15 (13%) had moderate-large urinary ketosis after 6 months [30]. Ketone levels do not always correlate with improved efficacy in adults [30, 33] or in mixed cohorts of children and adolescents [13, 23, 32].  Table 2 summarizes the studies of MAD in adults.

## 5. Other Uses of MAD

The MAD has rarely been used for other indications. Its successful use has been reported in Sturge-Weber syndrome [32] and GLUT1 deficiency syndrome [37]. Kumada et al. [38] reported resolution of nonconvulsive status epilepticus following administration of MAD to two children, one with frontal lobe epilepsy and the other with subcortical band heterotopias. Kossoff et al. reported use of MAD in adolescents with chronic daily headache. Only 3 patients out of 8 completed the three-month study. Two of them reported improvement in their headache severity [39].

## 6. Tolerability and Side Effects

The modified Atkins diet has generally been well tolerated. In the study by Kossoff et al., significant constipation was reported in four children [13]. Six children lost weight, median of 2.7 kg, three of whom were the heaviest in the cohort. Cholesterol values increased from a mean of 192 mg/dL to 221 mg/dL at the end of 6 months, but this increase was half of the reported increase with the ketogenic diet [40]. In the study on 30 adults who were administered the modified Atkins diet for refractory epilepsy, total cholesterol increased from 187 mg/dL to 201 mg/dL, but the LDL, HDL, and triglycerides remained in average risk ranges [30].

Gastrointestinal side effects such as constipation and vomiting have been the commonest side effects reported [12–14, 18, 30, 31]. These are most marked at the initiation of the diet, with time they improve [31]. Weight loss is prominent in obese patients [30]. One child developed aspiration pneumonia in the study by Kang et al. [14]. Kidney stones, seen in 5% of patients on the ketogenic diet, have not been reported with the modified Atkins diet to date.

## 7. Conclusion

MAD is an efficacious therapy for patients with refractory epilepsy. It is less restrictive and more palatable than the classical ketogenic diet. There is limited data regarding the efficacy of MAD with respect to the classical ketogenic diet. However, MAD is a prudent therapeutic option especially for older children and adolescents as it is a more “liberalized” diet as compared to classical ketogenic diet. It is also a good option for resource-constraint settings with paucity of trained dieticians.

## Figures and Tables

**Table 1:** Summary of studies involving children treated with MAD.

Authors	Study type	Sample size	Age range	Epilepsy	At 3 months		Adverse effects
					>50%	>90%	
Kossoff et al. 2003 [12]	Open trial	6	7–52 yrs	Mixed	3	—	Not reported
Kossoff et al. 2006 [13]	Prospective	20	3–18 yrs	≥3 Sz/week on at least 2 AEDs	50%	11%	Constipation
Kossoff et al. 2007 [18]	Randomized cross-over (10 g versus 20 g)	20	3–18 yrs	At least daily countable seizures	10 g—60% 20 g—10%	10 g—30% 20 g—0%	Improved tolerability with 20 g
Kang et al. 2007 [14]	Prospective	14	Mean 7.4 yrs	≥4 Sz/month; ≥3 AEDs	50%	29%	Gastrointestinal disturbances
Porta et al. 2009 [19]	Retrospective	27	4–182 months	≥2 Sz/week, ≥3 AEDs	KD—64% MAD—20%	—	Mild digestive disorders
Weber et al. 2009 [20]	Prospective	15	2–17 yrs	≥1 Sz/week, ≥2 AEDs	40%	13%	Not significant
Tonekaboni et al. 2010 [21]	Prospective	27	1–16 yrs	≥3 AEDs tried	67%	25%	Minor
Miranda et al. 2010 [22]	Prospective	33	1–16 yrs	Medically intractable epilepsy	52%	42%	Subtle
Kossoff et al. 2011 [23]	Prospective (MAD + 1 month Ketocal)	30	3–18 yrs	At least daily countable seizures; ≥2 AEDs	At 1 month, 80% At 2 months, 70%	37% 43%	Constipation
Groomes, et al. 2011 [24]	Retrospective + prospective	21 (8-ketogenic diet, 13-MAD)	Median age at diet onset—6 yrs	Intractable childhood and juvenile absence epilepsy	82%	38%	Not mentioned
Kumada et al. 2012 [25]	Prospective	10	1.5–17 yrs	≥3 Sz/week, ≥3 AEDs	At 3 weeks, 3	2	
Sharma et al. 2012 [26]	Prospective	15	6 m–3 yrs	Daily infantile spasms		40% spasm free	Constipation
Kim et al. 2012 [27]	Retrospective	20	21 m–17 yrs	Mixed	55%*	35%*	Constipation, hypercalciuria, hyperuricemia, transient lipase elevation
Sharma et al. 2013 [28]	Randomized controlled trial	Total 102, 50 randomized to diet group	2–14 yrs	Daily seizures, or at least 7/week	52% in diet group versus 11.5% in control group	30% in diet group versus 7.7% in control group	Constipation, anorexia, vomiting, lethargy

*Efficacy during the recent or final 3 months of the diet therapy.

AEDs: antiepileptic drugs; Sz: seizures; MAD: modified Atkins diet; KD: ketogenic diet.

**Table 2:** A summary of studies that include data specifically on individuals aged >12 years on MAD [29].

Study	Study design	Sample size (adolescents/adults)	Age (yrs)	Seizure types	Endpoint (mths)	Number of adolescent (12–18 yrs) responders (%) (>50% reduction)	Number of adult (>18 yrs) responders at endpoint (%) (>50% reduction)	Adverse effects
Kang et al. 2007 [14]	Prospective	1 (1/0)	14.4	DS with ATS	7	0 (0%)	NA	Vomiting
Kossoff et al. 2008 [30]	Prospective	30 (0/30)	18–53	CPS, MST, AS	6	NA	9 (30%)	Lethargy, weight loss, elevated total cholesterol, leg swelling
Carrette et al. 2008 [31]	Prospective	8 (0/8)	31–55	CPS, CPS with occasional SG, LGS	6	NA	1 (13%)	Vomiting, headache, nausea, diarrhoea, constipation, weakness, weight loss, elevated total and LDL cholesterol
Weber et al. 2009 [20]	Prospective	7 (7/0)	12–17	SFE, LGS, MAE, JME	3	3 (43%)	NA	Unknown
Kossoff et al. 2010 [32]	Prospective	2 (2/0)	13–18	SWS with CPS	6	1 (50%)	NA	Weight loss, high peak total cholesterol
Smith et al. 2011 [33]	Prospective	18 (0/18)	18–55	PS with SG, MS, CPS, SPS	12	NA	3 (17%)	Weight loss

AS: absence seizures; ATS: atonic seizures; CPS: complex partial seizures; DS: Doose syndrome; JME: juvenile myoclonic epilepsy; LGS: Lennox-Gastaut syndrome; MAE: myoclonic astatic epilepsy; MS: myoclonic seizures; MST: multiple seizure types; NA: not available; PS: partial seizures; SFE: symptomatic focal epilepsy; SG: secondary generalization; SPS: simple partial seizures; SWS: Sturge-Weber syndrome.
